# Medial Sigmoid Depression of the Mandibular Ramus as a Lesion-Mimicking Anatomical Variation: A Systematic Review

**DOI:** 10.3390/ijerph18084271

**Published:** 2021-04-17

**Authors:** Andy Wai Kan Yeung, Natalie Sui Miu Wong

**Affiliations:** 1 Oral and Maxillofacial Radiology, Applied Oral Sciences and Community Dental Care, Faculty of Dentistry, University of Hong Kong, Hong Kong, China; 2 Oral and Maxillofacial Surgery, Faculty of Dentistry, University of Hong Kong, Hong Kong, China; smwong26@hku.hk

**Keywords:** dental radiology, panoramic radiograph, anatomical variation, lesion, radiographic diagnosis, prevalence, diagnostic imaging

## Abstract

(1) Background: Medial sigmoid depression (MSD) of the mandibular ramus is an anatomical variation that resembles non-odontogenic cystic lesion. (2) Aim: The aim of this systematic review was to survey the literature to identify the relevant journal publications, reveal their scientific impact in terms of citations and compare the reported prevalence of MSD. (3) Materials and methods: PubMed, Google Scholar, Scopus and Web of Science were queried to identify relevant publications. The search string was: “medial depression of mandibular ramus” OR “medial depression of the mandibular ramus” OR “medial depression of the mandibular rami” OR “medial depression of mandibular rami” OR “medial sigmoid depression”. (4) Results: Eight studies were identified. Dry mandibles and patient dental panoramic radiographs were evaluated in four and seven of the eight studies, respectively. The prevalence of MSD varied from 20.2% to 82.0%. In male and female patients, the prevalence was 18.3–76.0% and 22.0–64.0%, respectively. MSD tended to occur bilaterally and most prevalent in patients with Angle’s Class II occlusion. The semilunar and triangular shapes were more common than teardrop and circular shapes. The most cited study had 12 citations. (5) Conclusions: MSD was a seldom investigated and cited anatomical variation that was not uncommon. Its recognition should be further promoted.

## 1. Introduction

**Figure 1:**
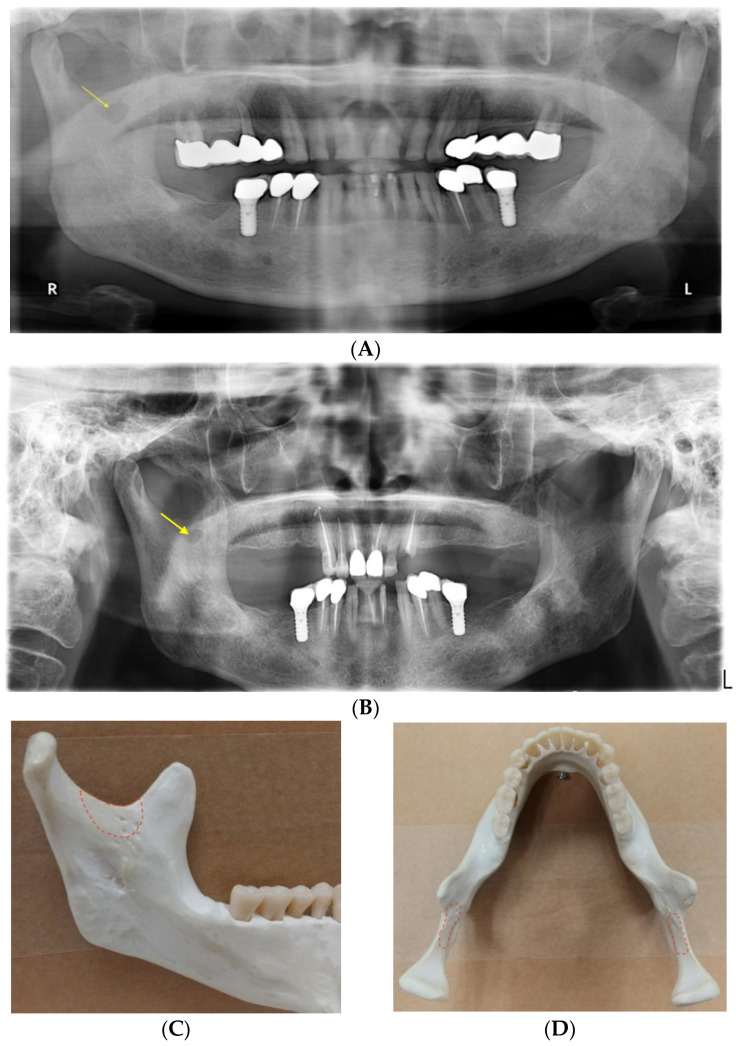
Dental panoramic radiographs showing a medial sigmoid depression (MSD) on the right side of the mandible, taken in (**A**) 2012 and (**B**) 2021, respectively. Its appearance did not change much between two timepoints. A mandible model showing the location where an MSD may be present from the (**C**) lingual and (**D**) axial views.

First described by Langlais et al. in 1983, the medial sigmoid depression (MSD) is a concavity located medial to the sigmoid notch of the mandible, present in over half of the evaluated samples [1]. Nearly four decades have lapsed since the publication of this pioneer work in the “Triple O” journal (known as Oral Surgery, Oral Medicine, Oral Pathology at that time). This anatomical entity was not indexed by Terminologia Anatomica and common dental radiology textbooks such as the ones by Whaites and Drage [2], White and Pharaoh [3] and Koenig et al. [4]. On a dental panoramic radiograph, the medial sigmoid depression may present as a radiolucent lesion inferior to the sigmoid notch that may or may not be connected to the latter (Figure 1A). The radiolucency is caused by reduced absorption of radiation due to the thinning of the bone on the lingual side of the sigmoid notch [5] (Figure 1B,C). The shape of MSD can be mainly classified into tear-drop, semilunar, circular and triangular [5]. The radiolucency may resemble a mandibular non-odontogenic cystic lesion that could lead to a dentist referring the patient to receiving additional advanced three-dimensional imaging with increased radiation dose. On the other hand, dentists are much more aware of the Stafne cavity (also known as Stafne cyst, defect or lacuna) that presents as a mandibular bone depression commonly located in the lingual posterior region of the mandible [6]. A common belief is that a hyperplastic or hypertrophic lobe of the major salivary glands may exert pressure upon the cortex of the mandible and result in the focal bone resorption [6]. Because of this mechanism, a Stafne cavity is usually slow-growing and apparent on radiographs of elderly patients. On the contrary, the MSD seemed to be much less recognized by the dental profession. The recognition of this anatomical entity, therefore, may save patients from receiving unnecessary radiation. The aim of this systematic review was to survey the literature to identify the relevant journal publications, reveal their scientific impact in terms of citations and compare the reported prevalence of MSD.

## 2. Materials and Methods

On 15 March 2021, four literature databases, namely PubMed, Google Scholar, Scopus and Web of Science, were queried. The following phrases were typed into the search string: “medial depression of mandibular ramus” OR “medial depression of the mandibular ramus” OR “medial depression of the mandibular rami” OR “medial depression of mandibular rami” OR “medial sigmoid depression”. These terms were entered in English only. For PubMed, Scopus and Web of Science, the search covered “All fields” instead of limiting to article title, abstract and keywords. All publications returned by the searches were initially included. Exclusion criteria included duplicate publications, irrelevance, no access and no reporting of the prevalence of MSD.

**Figure 2:**
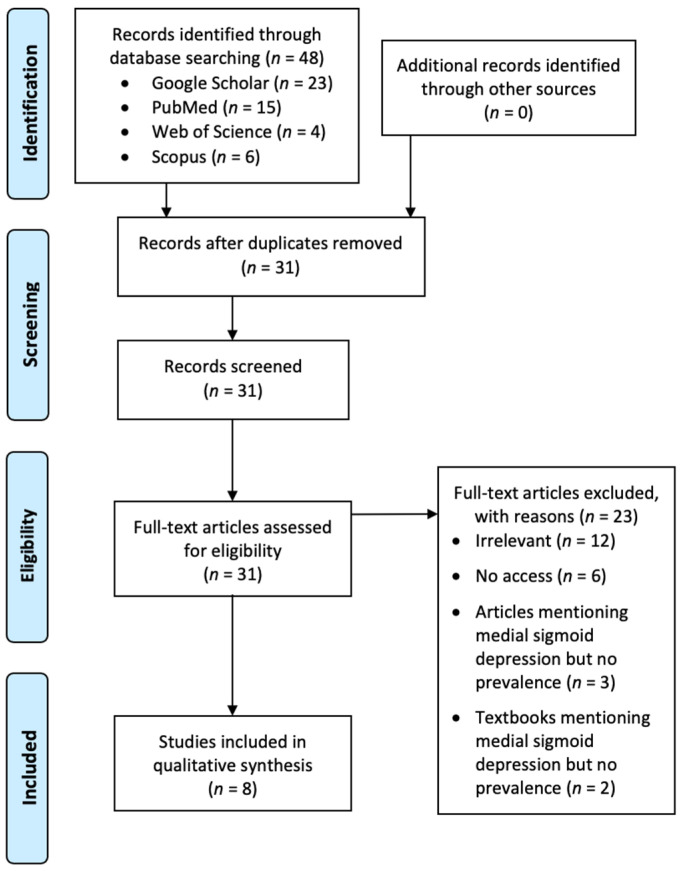
A Preferred Reporting Items for Systematic Reviews and Meta-Analyses (PRISMA) flow chart showing the screening process of the literature search.

The searches initially returned with 48 publications. After removing duplicates, 31 publications remained. After screening and excluding unsuitable publications with specific reasons, eight studies remained for the review (Figure 2). Each author did the screening independently and a final consensus was reached.

Ethical approval was not applicable to this review.

## 3. Results

The eight identified studies are listed in Table 1 [1,5,7,8,9,10,11,12]. The main text of two of them were not written in English, one of which was translated into English successfully by Google Docs [11], whereas the other one had extractable data from the abstract written in English [9]. One study investigated dry mandibles only, four studies investigated panoramic radiographs only and three studies investigated both. The prevalence of MSD varied from 20.2% to 82.0%. In male and female patients, the prevalence was 18.3–76.0% and 22.0–64.0%, respectively. Most studies reported that MSD tended to occur bilaterally. Three studies reported that MSD was most prevalent in patients with Angle’s Class II occlusion (meaning that the maxillary first molar is positioned more anteriorly than normal relative to the mandibular first molar), whereas one study reported that it was most prevalent in Class I occlusion (normal occlusion). The semilunar and triangular shapes were more common than teardrop and circular shapes.

Data from Google Scholar indicated that the most cited paper was Langlais et al. [1] with 12 citations, followed by Carvalho et al. [5] with 8 citations. Other articles had 0–3 citations. No additional suitable paper was identified from these citing papers.

## 4. Discussion

This systematic review identified eight studies that reported the prevalence of MSD in dry mandibles and dental panoramic radiographs. They reported a wide range of overall prevalence as well as the prevalence in various subgroups according to sex, side of the mandible, Angle’s classification of occlusion and shape. There seemed to be no sex bias in having MSD. All reviewed studies received few citations. Langlais et al. [1] was acknowledged by four of the evaluated studies as the first report of MSD and it was otherwise cited in the introduction by the remaining three studies. Readers should be aware that the highest prevalence of MSD reported from dental panoramic radiographs was 70.0%, implying that MSD may be a commonly observed but seldom investigated anatomical entity. To support the “As Low As Reasonably Achievable” (ALARA) principle of using ionizing radiation [13], dentists and other healthcare workers should be able to recognize MSD on panoramic radiographs, so that unnecessary additional radiographic assessments would not be ordered for patients. Recently, the cone beam computed tomography (CBCT) has been more readily available and popular among dentists as it visualizes the region of interest in three-dimensional manner in high resolution. Its usage in various occasions for diagnosis and treatment planning in dental medicine has been advocated [14]. A thorough understanding of the radiographic anatomy and the radiographic device may avoid unnecessary CBCT examination with increased radiation dose [15,16].

Indeed, MSD was not mentioned in the most common dental radiology textbooks as mentioned in the Introduction. Five of the eight reviewed studies were published in journals without an impact factor. However, other textbooks did mention it, such as Wood and Goaz [17] and William Jr and Merritt [18]. MSD was also briefly mentioned by several other papers in the literature as a potential radiographic finding or a differential diagnosis for other pathologies [19,20,21,22,23]. One common differential diagnosis would be Stafne cavity, which usually occurs at the angle of the mandible below the mandibular canal but can also occur at other locations in the mandible [6]. It should be noted that Stafne cavity had a strong male preponderance [6] but MSD did not. In addition, it could be potentially referred to or discussed with other names, such as pseudocyst in the coronoid process [24] and coronoid foramina or foramen [25,26].

There were some limitations of this review. First, only eight studies were eligible to be reviewed. This small sample of papers might not be enough to obtain a very precise conclusion regarding the prevalence of MSD. Second, there was a wide range of the number of panoramic radiographs examined by the reviewed studies, rendering them not very homogeneous.

## 5. Conclusions

To conclude, MSD was a seldom investigated and cited anatomical variation. It has a high relevance to oral and maxillofacial radiology and head and neck imaging, as its radiographic appearance resembles a non-odontogenic cystic lesion in the mandible. Its recognition should be further promoted. Based on very limited data available from dry mandible and patient radiographic studies, its prevalence was reported to be from 20.2% to 82.0%.

## Figures and Tables

**Table 1:** Details of the eight included studies of medial sigmoid depression (MSD).

											Prevalence						
Reference	Year	No. of Dry mandibles	No. of Patient Panoramic Radiographs	No. of MSD Reported	MSD	Male	Female	Unilateral MSD ^1^	Bilateral MSD ^1^	Angle Class I	Angle Class II	Angle Class III	Tear-drop ^2^	Semilunar ^2^	Circular ^2^	Triangular ^2^	No. of Citations
[11]	2020	/	1000	298	0.234	0.220	0.242	0.170	0.064				0.289	0.326	0.077	0.309	0
[8]	2019	50		76	0.820			0.120	0.700				0.158	0.342	0.053	0.447	1
			50	64 ^3^	0.700	0.760	0.640	0.160	0.540				0.094	0.641	0.078	0.188	
[7]	2018	/	110	83	0.500					0.525	0.484	0.487	0.120	0.361	0.289	0.229	2
[12]	2014	/	300	106 ^3^	0.233			0.117	0.117	0.190	0.280	0.230	0.210	0.343	0.171	0.286	3
[9]	2003	/	465							0.242 ^4^	0.389 ^4^	0.313 ^4^					2
[5]	2001	251		118	0.339			0.131	0.208								8
			2067	586	0.202	0.183	0.220	0.120	0.082	0.124	0.329	0.321	0.200	0.314	0.089	0.397	
[10]	1991	78	/					0.282	0.333								2
[1]	1983	88		76	0.557			0.250	0.307								12
			1986	226	0.082			0.050	0.032								

^1^ Prevalence based on the total number of reported patients. ^2^ Prevalence based on the total number of reported MSD. ^3^ The study reported conflicting total numbers of MSD in patients. The largest number was listed here. ^4^ Values computed by simple averaging of data reported for right and left sides for each Class, respectively.

## Data Availability

Not applicable.
